# Low-dose dasatinib in second-line therapy or beyond for the treatment of chronic-phase chronic myeloid leukemia: a real-world cohort study

**DOI:** 10.3389/fphar.2026.1734319

**Published:** 2026-06-18

**Authors:** Yilin Chen, Hongxiang Wang, Fang Cheng, Weiming Li

**Affiliations:** 1 Department of Hematology, The First Affiliated Hospital of Zhengzhou University, Zhengzhou, China; 2 Department of Hematology, Union Hospital, Tongji Medical College, Huazhong University of Science and Technology, Wuhan, China; 3 Department of Hematology, The Central Hospital of Wuhan, Wuhan, China; 4 Department of Pharmacy, Union Hospital, Tongji Medical College, Huazhong University of Science and Technology, Wuhan, China

**Keywords:** chronic myeloid leukemia, dasatinib, later-line, molecular response, safety, TKI dose optimization

## Abstract

**Background:**

Dasatinib is a favorable option for patients with chronic-phase chronic myeloid leukemia (CML-CP) who have an inadequate response, treatment intolerance, or are attempting treatment-free remission (TFR). Dasatinib dose reduction is a strategy to improve tolerability while maintaining efficacy in first-line settings or patients with a stable molecular response. However, whether low-dose dasatinib is safe and effective in second-line or later setting for patients with CML-CP remains unclear.

**Methods:**

We conducted a retrospective analysis of 53 patients with CML-CP who switched to low-dose dasatinib due to inadequate molecular response (n = 37), treatment intolerance (n = 13), or to re-induce a deeper molecular response (n = 3).

**Results:**

Among the 37 patients who had not achieved major molecular response (MMR) before switching, 10 patients (27.0%) attained MMR, 12 patients (32.4%) achieved deep molecular response (DMR/MR4), and 11 patients (29.7%) reached molecular response 4.5 (MR4.5) following low-dose dasatinib therapy. Notably, 81.8% of patients with only baseline MMR improved to MR4 or MR4.5 after switching to low-dose dasatinib. One patient lost MR4 while receiving dasatinib at 50 mg/day and subsequently switched to nilotinib. All three patients who were switched to re-induce deeper molecular responses successfully re-attained MR4.5 after switching to low-dose dasatinib. Adverse events resolved in all 13 patients who switched due to prior TKI intolerance, and no worsening grade 2 or more adverse events were observed.

**Conclusion:**

Our findings suggest that low-dose dasatinib for selected patients with CML-CP in second-line therapy or beyond may be safe and effective under close monitoring. Nevertheless, multicenter prospective clinical trials are needed to validate the efficacy and safety of low-dose dasatinib therapy in second-line treatment settings or beyond in CML.

## Introduction

The introduction of tyrosine kinase inhibitors (TKIs) has revolutionized the treatment landscape of chronic-phase chronic myeloid leukemia (CML-CP), allowing most CML-CP patients to achieve near-normal life expectancy (Bower et al., 2016). However, chronic adverse effects of TKIs such as fatigue, pleural effusion, and metabolic disorders could frequently impair patients’ quality of life and mental health. Moreover, the cumulative financial burden of long-term treatment remains substantial, particularly in healthcare settings with limited reimbursement (Winn et al., 2016; Jiang et al., 2017; Efficace et al., 2021). Although treatment-free remission (TFR) is an important therapeutic goal, only 30%-50% of patients meet eligibility criteria for TKI discontinuation. Of those who attempt discontinuation, approximately half relapses and require treatment reinitiation (Saussele et al., 2018; Kantarjian et al., 2021). As a result, nearly 70% of patients remain on indefinite TKI therapy (Baccarani and Gale, 2021).

TKI dose optimization has emerged as a practical approach to improve tolerability, reduce toxicity, and alleviate economic pressure. Low-dose TKI therapy has shown favorable efficacy in maintaining molecular responses in CML patients with stable remission (Chen et al., 2022). Furthermore, dose reduction of TKIs prior to discontinuation does not compromise the likelihood of TFR (Clark et al., 2019). Dasatinib, one of the second-generation TKIs (2G-TKIs), is particularly effective in CML patients who exhibit resistance or intolerance to previous TKI (Cortes et al., 2016). Specifically, dasatinib is effective in cases of the Y253H, E255K/V, or F359C/V mutation within the BCR::ABL1 kinase domain (Jabbour and Kantarjian, 2022). Nevertheless, long-term standard-dose administration can be poorly tolerated and financially burdensome.

Multiple studies have investigated the efficacy and safety of standard-dose dasatinib (100 mg/day) versus low-dose therapy (<100 mg/day) in both clinical trials and real-world settings. Naqvi and colleagues prospectively evaluated 83 CML patients receiving dasatinib 50 mg/day as first-line therapy. The cumulative rates for a major molecular response (MMR), a deep molecular response (DMR/MR4), and a molecular response 4.5 (MR4.5) by 12 months were 81%, 55%, and 49%, respectively (Naqvi et al., 2020). The clinical response and toxicity profile observed with half-dose dasatinib as initial therapy were more favorable compared with the outcomes reported in the DASISION trial (Cortes et al., 2016). Similarly, a propensity-score matched comparative study demonstrated that dasatinib at 50 mg/day was at least as effective as 100 mg daily in newly diagnosed CML patients, with superior safety profiles and drug exposure (Jabbour et al., 2022). In the DAVLEC study, 52 CML patients aged over 70 years received first-line treatment with dasatinib 20 mg/day, with 31 patients (60%) achieving MMR at 12 months (Murai et al., 2021).

Despite the promising findings described above, data on the use of low-dose dasatinib in second-line settings or beyond, particularly for CML patients who are switched to this reduced-dose regimen due to reasons such as inadequate response or intolerance to prior TKIs, is limited. Therefore, this retrospective study was conducted to evaluate the efficacy and safety of low-dose dasatinib in CML-CP patients in second-line therapy or beyond, aiming to provide real-world evidence to inform individualized dose optimization beyond the first-line setting.

## Materials and methods

### Patients

This retrospective, single-center study was conducted at Union Hospital, Tongji Medical College, Huazhong University of Science and Technology. Data were collected from patients with CML-CP who received low-dose dasatinib as second-line treatment or beyond between November 2021 and March 2025. All patients were diagnosed with CML-CP based on bone marrow morphology, cytogenetic analysis, and molecular testing. The inclusion criteria were: (1) a confirmed diagnosis of CML-CP; and (2) regular follow-up during low-dose dasatinib therapy. Patients were excluded if they were in the accelerated or blast phase, or if follow-up data were incomplete or irregular. Demographic and clinical data were extracted from the electronic medical record system, including sex, age at diagnosis, date of diagnosis, TKI type and dose, reasons for treatment modification, BCR::ABL1 levels, and treatment-emergent adverse events.

The recommended standard dose of dasatinib for CML-CP patients who had inadequate response or intolerance to prior TKIs is 100 mg/day. Low-dose dasatinib refers to a dose lower than the recommended dose, including 50 mg/day and 70 mg/day. Patients were switched to low-dose dasatinib for the following reasons: 1) suboptimal response to prior TKI therapy (classified as Warning or Failure according to ELN recommendations), defined by stable or rising BCR::ABL1 levels on at least two consecutive molecular assays; 2) intolerance to prior TKI therapy; or 3) failure to achieve MR4 despite prior therapy, with stable BCR::ABL1 levels on at least two assays and pursuing TFR. Switching to low-dose dasatinib was recommended by a single oncologist and was voluntarily accepted by the adult patients or by the legal guardians for those under 18 years of age. The switch to a 50 mg or 70 mg dose of dasatinib was determined based on the molecular response and the adverse events associated with the previous TKI treatment. Patients who received dasatinib at 50 mg once daily initiated treatment directly at this dose, rather than starting at 100 mg daily and subsequently undergoing dose reduction.

This study was approved by the Ethics Committee of Union Hospital, Tongji Medical College, Huazhong University of Science and Technology (2025 [0236]). Informed consent was waived due to the retrospective nature of the study.

### Efficacy

Patients included in this study were followed up during low-dose dasatinib therapy through molecular monitoring that was performed every 3 months. If the molecular response was inadequate and adverse events were tolerable, the dasatinib dose was escalated to 100 mg/day, or treatment was switched to another TKI. The primary endpoint for this study was the proportion of patients who reached MMR, MR4 and MR 4.5 on low-dose dasatinib treatment. Molecular monitoring was assessed by quantitative polymerase chain reaction (qPCR). The BCR::ABL1 level was expressed on the international scale (IS). The molecular response was assessed according to ELN criteria (Hochhaus et al., 2020). In detail, MMR was defined as a BCR::ABL1 ratio ≤0.1%, MR4 was defined as a BCR::ABL1 ratio ≤0.01%, and MR4.5 was defined as a BCR::ABL1 ratio ≤0.0032%, with ABL1 used as the internal reference control gene; a minimum of 32,000 ABL1 transcripts was required for MR4.5 assessment.

### Adverse events

Adverse events were graded according to National Cancer Institute Common Terminology Criteria for Adverse Events version 3.0.

### Statistical analysis

Continuous variables are reported as the median and the interquartile range (IQR) or mean ± standard deviation (SD). Cumulative response rates were calculated using the cumulative incidence approach and Kaplan-Meier method. The log-rank test was used for the comparison between groups. P < 0.05 was regarded as statistically significant. All statistical analyses were performed using the R software version 4.1.2 (R Foundation for Statistical Computing, Vienna, Austria).

## Results

### Patient characteristics

The study included 53 CML patients who switched to low-dose dasatinb therapy (Table1). The median ages at diagnosis and at switching to low-dose dasatinib were 39 (IQR: 27–50) and 42 (IQR: 33–51.5) years, respectively. At the time of low-dose dasatinib initiation, all patients were aged ≥14 years. Two patients were younger than 18 years, and both had body weights >45 kg. Thirty-five patients (66.0%) were females. The median duration of TKI treatment before switching to low-dose dasatinib was 30 months (IQR: 4.5–60). Most patients (75.5%) had received one TKI prior to switching to low-dose dasatinib, while 13 (24.5%) had been treated with two or three different TKIs. The main reason for switching was failure to achieve MMR or MR4 (n = 37, 69.8%), followed by TKI intolerance (n = 13, 24.5%) and attempt for TFR (n = 3, 5.6%). Among the 53 patients included in the study, 37 failed to achieve MMR, five patients achieved MR4, and 11 patients achieved MMR while on previous therapy. Of the five patients in MR4 at baseline, three switched after losing MR4.5 (while maintaining MR4) on imatinib in order to re-induce a deeper molecular response. The remaining two patients were switched to low-dose dasatinib therapy due to adverse effects: one for grade 2 imatinib-related edema and the other for grade 2 flumatinib-related hepatotoxicity. A description of continuous variables using both parametric and non-parametric statistics is shown in Supplementary Table S1. Median durations before switching to low-dose dasatinib were 23.5 months (IQR: 7–34) for MMR and 12 months (IQR: 3.5–72.5) for MR4. Median times to achieve MMR and MR4 before switching to low-dose dasatinib were 14.5 months (IQR: 6.25–21.5) and 37 months (IQR: 10.5–59.5), respectively. Median follow-up of patients after switching to low-dose dasatinib therapy was 21 months (IQR: 13.5–27). All patients received low-dose TKI therapy on a voluntary basis. Most patients (n = 51) received dasatinib at 50 mg/day. Two patients with prior TKI intolerance were switched to low-dose dasatinib at 70 mg/day due to concerns regarding suboptimal efficacy.

**TABLE 1:** Patient characteristics, TKI treatment, and molecular responses before switching to low-dose dasatinib.

Characteristic	N = 53
Age at diagnosis, years, median (IQR)	39 (27–50)
Age at diagnosis, n (%)	
≤20	7 (13.2)
20–40	19 (35.8)
40–60	19 (35.8)
>60	6 (11.3)
Age at switching to low-dose dasatinib, years, median (IQR)	42 (33–51.5)
Age at switching to low-dose dasatinib, n (%)	
≤20	4 (7.5)
20–40	19 (35.8%)
40–60	22 (41.5%)
>60	8 (15.1%)
Female, sex, n (%)	35 (66.0)
TKI treatment before low-dose dasatinib treatment, n (%)	
Imatinib	31 (58.4)
Nilotinib	10 (18.8)
Flumatinib	12 (22.6)
Duration of TKI treatment before switching to low-dose dasatinib, months, median (IQR)	30 (4.5–60)
Number of TKI(s) before low-dose dasatinib, n (%)	
1	40 (75.5)
2 or 3	13 (24.5)
Reasons for switching to low-dose dasatinib, n (%)	
Failure to achieve MMR or MR4	37 (69.8)
TKI intolerance	13 (24.5)
Re-induction of deeper molecular responses	3 (5.6)
Molecular response status before switching to low-dose dasatinib, n (%)	
MR4	5 (9.4)
Only in MMR	11 (20.8)
Not in MMR	37 (69.8)
BCR::ABL1 level before switching to low-dose dasatinib, n (%)	
≤0.1%	16 (30.2)
0.1%–1%	15 (28.3)
1%–10%	11 (20.7)
>10%	11 (20.7)
Duration of MMR before switching to low-dose dasatinib, months, median (IQR)	23.5 (7–34)
Duration of MR4 before switching to low-dose dasatinib, months, median (IQR)	12 (3.5–72.5)
Time to achieve MMR before switching to low-dose dasatinib, months, median (IQR)	14.5 (6.25–21.5)
Time to achieve MR4 before switching to low-dose dasatinib, months, median (IQR)	37 (10.5–59.5)
Follow-up of low-dose dasatinib, months, median (IQR)	21 (13.5–27)
Dasatinib dose, n (%)	
50 mg/day	51 (96.2)
70 mg/day	2 (3.8)

IQR, interquartile range; TKI, tyrosine kinase inhibitor; MMR, major molecular response; MR4, deep molecular response (DMR).

### Efficacy

Treatment regimens and molecular responses before and after low-dose dasatinib are summarized in Table 2. Among the 37 patients who did not achieve MMR at baseline, 10 patients (27.0%) achieved MMR, 12 patients (32.4%) achieved MR4, and 11 patients (29.7%) achieved MR4.5 after switching to low-dose dasatinib therapy. However, 4 patients (10.8%) among those who had not reached MMR at baseline also failed to achieve MMR after switching to low-dose dasatinib therapy. The median time to MMR was 4 months (IQR: 3–9.5) in patients using the low-dose dasatinib therapy, with a median MMR duration of 17 months (IQR: 10.5–23). The median time to achieve MR4 was 9 months (IQR: 5–18), with a median duration of 9 months (IQR: 3–19). For MR4.5, the median time to response was 12 months (IQR: 5–15), with a median duration of 11 months (IQR: 10–21). Among the 37 patients who had not achieved MMR prior to switching, the baseline BCR::ABL1 levels were distributed as follows: 0.1%-1% (n = 15), 1%-10% (n = 11), and >10% (n = 11). Treatment responses across the three baseline BCR::ABL1 groups are shown below: In the group with BCR::ABL1 levels of 0.1%-1%, 14 of 15 patients (93.3%) showed an improved molecular response. Similarly, 9 of 11 patients (81.8%) with baseline levels of 1%-10% demonstrated improvement. Among the 11 patients with baseline BCR::ABL1 levels over 10%, 10 patients (90.9%) experienced improvement in molecular response. Among the 37 patients without MMR at baseline, 11 switched to low-dose dasatinib primarily because of intolerance to prior TKI therapy, whereas 26 switched because of inadequate molecular response. According to the ELN criteria, of the 26 patients, 19 were classified as “warning” and 7 as “unfavorable”. After switching to low-dose dasatinib, in the warning group, 6 patients achieved MMR, 6 achieved MR4, and 6 reached MR4.5. In the unfavorable group, 2 patients achieved MMR, one achieved MR4, and 2 reached MR4.5.

**TABLE 2:** Treatment regimens and molecular response before and after low-dose dasatinib treatment.

Baseline response	Last TKI before low-dose dasatinib	Median TKI duration before low-dose dasatinib, (IQR), months	Number of TKI(s) before low-dose dasatinib	Response after low-dose dasatinib
Not in MMR (n = 37)	Imatinib 400 mg (n = 22)	5 (0.6–62)	1 (n = 22)	Not in MMR (n = 1) MMR (n = 6) MR4 (n = 8) MR4.5 (n = 7)
	Flumatinib 600 mg (n = 8)	10 (3.2–20.5)	1 (n = 6) 3 (n = 2)	Not in MMR (n = 2) MMR (n = 1) MR4 (n = 2) MR4.5 (n = 3)
	Nilotinib 300 mg bid (n = 7)	69 (29–86)	1 (n = 2) 2 (n = 5)	Not in MMR (n = 1) MMR (n = 3) MR4 (n = 2) MR4.5 (n = 1)
Only in MMR (n = 11)	Imatinib 400 mg (n = 5)	37 (18–58)	1 (n = 5)	MMR (n = 1) MR4 (n = 3) MR4.5 (n = 1)
	Flumatinib 600 mg (n = 3)	34 (30–48)	1 (n = 1) 2 (n = 2)	MMR (n = 1) MR4 (n = 1) MR4.5 (n = 1)
	Nilotinib 300 mg bid (n = 3)	50 (30–73)	2 (n = 3)	MR4 (n = 3)
​	Imatinib 400 mg (n = 4)	39.5 (30–154)	1 (n = 4)	Lost MR4 (n = 1) MR4.5 (n = 3)
MR4 (n = 5)	Flumatinib 600 mg (n = 1)	13	1 (n = 1)	MR4.5 (n = 1)

IQR, interquartile range; TKI, tyrosine kinase inhibitor; MMR, major molecular response; MR4, deep molecular response (DMR); MR4.5, molecular response 4.5.

Among 11 patients who had MMR but not MR4 at baseline, 7 patients (63.6%) reached MR4, 2 patients (18.2%) achieved MR4.5, while 2 patients (18.2%) remained in MMR. The median time to MR4 was 4 months (IQR: 3–14), with a median duration of 14 months (IQR: 4–17). Of the five patients in MR4 at baseline, 4 patients (80.0%) achieved MR4.5. The median time to MR4.5 was 9 months (IQR: 3–12), with a median duration of 11 months (IQR: 6–23.5). One patient lost MR4 to MMR after 9 months on dasatinib 50 mg/day and was therefore switched to nilotinib.

In the entire cohort of 53 patients, 37 patients (69.8%) had not achieved MMR, 11 patients (20.8%) were in MMR, and five patients (9.4%) were in MR4 at baseline before switching to low-dose dasatinib treatment. Following the transition to low-dose dasatinib, 12 patients (22.6%) maintained MMR, 19 patients (35.8%) achieved MR4, and 17 patients (32.1%) achieved MR4.5. Four patients (7.5%) did not achieve MMR and required additional treatment adjustment, and one patient (1.9%) experienced loss of MR4.

**FIGURE 1:**
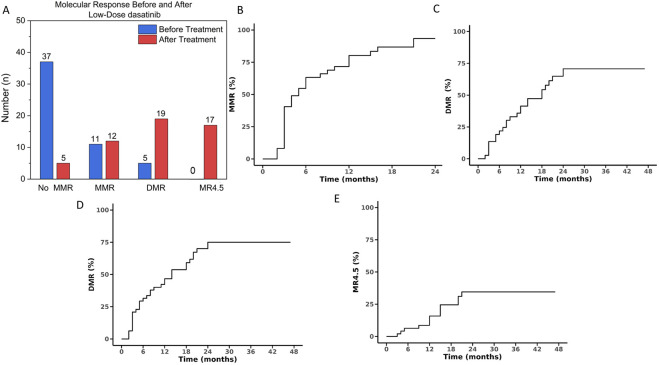
The molecular response in CML-CP patients at baseline and after switching to low-dose dasatinib therapy. **(A)** The molecular response Before and After low-dose dasatinib. **(B)** The cumulative MMR rate in 37 patients without MMR. **(C)** The cumulative MR4 rate in patients without MMR. **(D)** The cumulative MR4 rate in patients without MR4. **(E)** The cumulative MR4.5 rate in patients without MR4.5. MMR, major molecular response; MR4, deep molecular response (DMR); MR4.5, molecular response 4.5.

The molecular response before and after the switch to low-dose dasatinib is shown in Figure 1A. Among 37 patients who had not achieved MMR, the 2-year cumulative rates of MMR and MR4 were 93.4% (95% CI: 73.2%-98.5%) and 70.7% (95% CI: 48.0%-84.9%), respectively (Figures 1B,C). Among 48 patients who had not achieved MR4 at baseline, the 2-year cumulative rates of MR4 and MR4.5 were 75.0% (95% CI: 55.4%-87.0%) and 34.6% (95% CI: 19.3%-50.3%), respectively (Figures 1D,E). Given a median follow-up of 21 months, longer follow-up is required to confirm the long-term efficacy of low-dose dasatinib.

### Subgroup analysis

Among 40 patients who had received only one TKI before switching to low-dose dasatinib, 35 patients (87.5%) experienced improved molecular response. Among patients who were treated with more than one TKI before switching, 12 patients (92.3%) showed improved molecular response. Regarding prior therapy, improved responses were seen in 28 of 31 patients (90.3%) previously on imatinib and in 19 of 22 patients (86.4%) previously on 2G-TKIs.

Among the 37 patients without MMR at baseline, the cumulative incidence rates of MMR and MR4 following low-dose dasatinib were not significantly different between those who received one TKI and those who received more than one TKI before.

**FIGURE 2:**
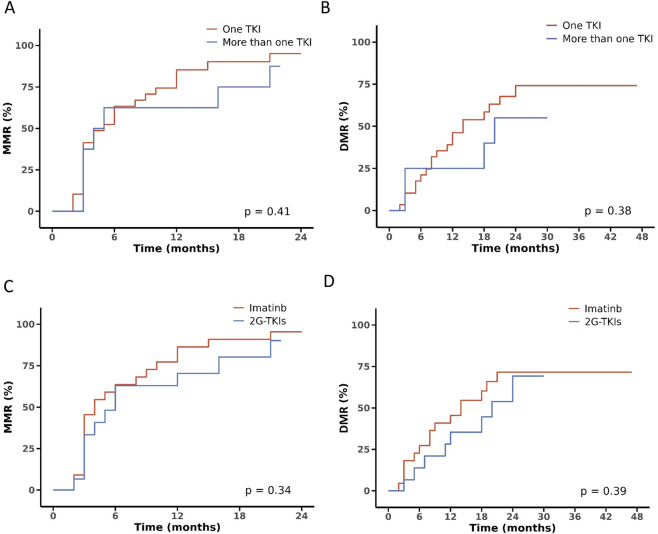
Cumulative incidence of molecular responses following low-dose dasatinib in patients without MMR at baseline, stratified by prior TKI treatment. **(A)** MMR by number of prior TKIs. **(B)** MR4 by number of prior TKIs. **(C)** MMR by type of immediately administered TKI before low-dose dasatinib. **(D)** MR4 by type of immediately administered TKI before low-dose dasatinib. TKI, tyrosine kinase inhibitor, MMR, major molecular response; MR4, deep molecular response (DMR).

Switching to low-dose dasatinib therapy (MMR, P = 0.41; MR4, P = 0.38; Figures 2A,B, respectively). Similarly, no significant differences in MMR or MR4 rates were observed between patients whose prior therapy was imatinib versus a 2G-TKI (MMR, P = 0.34; MR4, P = 0.39; Figures 2C,D, respectively).

**FIGURE 3:**
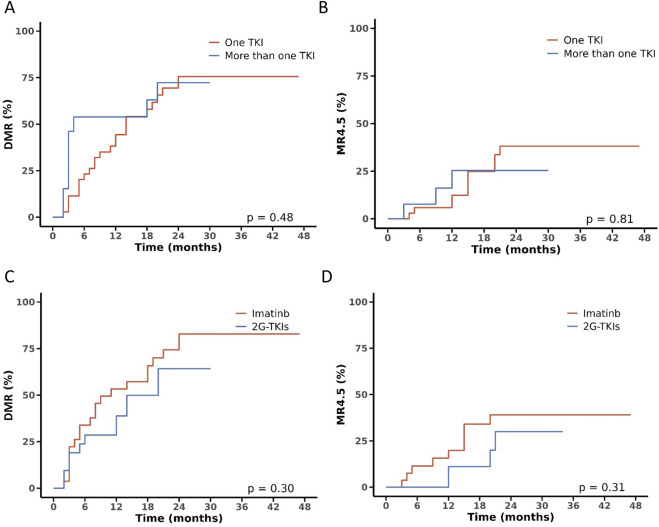
Cumulative incidence of molecular responses following low-dose dasatinib in patients without MR4 at baseline, stratified by prior TKI treatment. **(A)** MR4 by number of prior TKIs. **(B)** MR4.5 by number of prior TKIs. **(C)** MR4 by type of immediately administered TKI before low-dose dasatinib **(D)** MR4.5 by type of immediately administered TKI before low-dose dasatinib. TKI, tyrosine kinase inhibitor; MR4, deep molecular response (DMR); MR4.5, molecular response 4.5.

Among the 48 patients who had not achieved MR4 at baseline, no significant differences were observed in the cumulative incidences of MR4 or MR4.5 between patients who were treated with one prior TKI and those treated with 2 or more prior TKIs (MR4, P = 0.48; MR4.5, P = 0.81; Figures 3A,B, respectively). Similarly, the MR4 and MR4.5 rates were comparable between patients whose immediate prior therapy was imatinib versus 2G-TKIs (MR4, P = 0.30; MR4.5, P = 0.31; Figures 3C,D, respectively).

### Adverse events after switching to low-dose dasatinib

Thirteen patients switched to low-dose dasatinib due to intolerance to prior TKI therapy. Eleven patients transitioned from imatinib to low-dose dasatinib, most commonly because of edema (n = 3, 27.3%), gastrointestinal discomfort (n = 2, 18.2%), or fatigue (n = 2, 18.2%). Most of these adverse events were grade 1 (n = 9) or grade 2 (n = 2), and all resolved after switching. Two patients receiving flumatinib switched to low-dose dasatinib due to grade 1 diarrhea and grade 2 liver function abnormalities, both of which resolved after the switch.

Low-dose dasatinib was generally well tolerated, with no hematologic adverse events observed. Non-hematologic adverse events, all grade 1, included rash (n = 3, 5.7%), pleural effusion (n = 1, 1.9%), diarrhea (n = 1, 1.9%), poor appetite (n = 1, 1.9%), abnormal liver function (n = 1, 1.9%), and elevated uric acid (n = 1, 1.9%).

## Discussion

Dose reduction has been proposed as a feasible strategy to improve treatment adherence and reduce toxicity, without compromising efficacy. Low-dose dasatinib has been shown to be safe and effective for patients with newly diagnosed CML-CP (Jabbour et al., 2022). Previous studies have investigated outcomes of patients with stable molecular responses after TKI dose reduction, suggesting that low-dose TKI can effectively maintain treatment response (Rea et al., 2017; Cayssials et al., 2018; Li et al., 2025). In our previous study, we observed that TKI dose reduction due to drug intolerance among 10 CML-CP patients who did not reach MMR resulted in improved response as one patient achieved MMR, and six reached MR4 (Chen et al., 2022). In the study by Claudiani et al., where all patients had baseline MMR, 6 of 31 patients without MR4 achieved a deeper molecular response with low-dose dasatinib treatment (Claudiani et al., 2021). These studies indicate that low-dose TKI treatment, including dasatinb, does not impair subsequent MR4 achievement. Moreover, switching to dasatinib therapy is appropriate in patients who could not tolerate other TKIs due to hematologic and/or non-hematologic adverse events as well as in patients who develop TKI resistance, particularly those harboring BCR::ABL1 kinase domain mutations known to be sensitive to dasatinib (Jabbour and Kantarjian, 2022). Nevertheless, information regarding the use of low-dose dasatinib in the second-line treatment settings or beyond in CML-CP patients is lacking. Therefore, our study aimed to provide preliminary insights into the safety and efficacy of low-dose dasatinib in a real-world cohort of CML-CP patients who had failed or were intolerant to prior TKI(s).

In this study, among the 37 patients who had not achieved MMR prior to low-dose dasatinib, 33 patients (89.2%) achieved a deeper molecular response after switching, including 10 achieving MMR, 12 achieving MR4, and 11 achieving MR4.5. Notably, the majority of patients who achieved MMR at baseline were able to reach a deeper molecular response after switching to low-dose dasatinib (81.8%). The improved response rate observed in this study may be attributable to the small proportion of patients with baseline BCR::ABL1 levels >10%. In a pilot study by Iriyama et al., nine patients with imatinib-resistance received reduced-dose dasatinib at 50 mg/day. Among the eight patients who did not reach MMR, four patients subsequently achieved MMR, and one patient who had already reached MMR further achieved MR4 following low-dose dasatinib treatment (Iriyama et al., 2018). The improved response rate observed in our study, however, appears higher than that reported by Iriyama et al., potentially attributable to the inclusion of patients with prior intolerance to TKIs. In a study of low-dose ponatinib treatment for 52 patients with TKI intolerance, 21 patients demonstrated improved treatment response following low-dose ponatinib treatment (Iurlo et al., 2020). These findings suggest that molecular response can be further improved even with reduced doses of 2G-TKIs and third-generation TKIs in a subset of patients who had not achieved adequate response on standard dosing. Fassoni et al. developed a mathematical model based on real-world data from patients in the IRIS and CML-IV studies, describing the time course of TKI response as a dynamic process (Fassoni et al., 2018). The model indicates that long-term treatment response depends on the rare activation of quiescent leukemia stem cells. TKI efficacy requires maintaining therapeutic plasma concentrations within a specific range, with the predicted minimum effective dose at 25% of the original dose. The enhanced efficacy observed with low-dose TKI may be attributed to maintenance of effective plasma concentrations at the reduced dosages. In addition, recent studies suggest that after achieving DMR, TKI de-escalation may not reduce anti-leukemic activity against residual leukemic stem cells in most patients and may even enhance sensitivity to TKI treatment by inducing leukemic stem-cell cycling, which could potentially favor subsequent TFR in selected patient (Laganà et al., 2024). Given that our median follow-up period is 21 months, further follow-up is needed to validate the long-term efficacy and safety of low-dose dasatinib. Despite the promising findings, low-dose TKI therapy carries inherent risks, including loss of response to treatment, disease relapse, and the development of drug resistance. In the study by Claudiani et al. described earlier, among 246 patients receiving low-dose TKI, one progressed to the accelerated phase, and two developed novel ABL kinase domain mutations (T315I and V299L), demonstrating that disease progression and resistance, though infrequent, remain possible under reduced dosing (Claudiani et al., 2021). Together, these findings emphasize the need for careful monitoring for patients using low-dose TKIs to assure the efficacy and safety of treatment.

Compared with first-line imatinib, first-line nilotinib or dasatinib is associated with a higher rate of TFR eligibility, largely because these 2G-TKIs induce faster and deeper molecular responses (Fava et al., 2019; Etienne et al., 2020). Current evidence suggests that the probability of maintaining TFR is determined mainly by treatment duration and sustained DMR rather than by TKI generation itself (Mahon et al., 2024; Zheng et al., 2024; Laganà et al., 2025). Our previous work has shown that patients who switched to 2G-TKIs due to imatinib resistance or intolerance achieved TFR rates comparable to those receiving 2G-TKIs as frontline therapy (Chen et al., 2023). Similarly, comparable 48-week TFR rates were observed in patients receiving nilotinib as first- and second-line therapy (58% vs. 51.6%) (Mahon et al., 2018; Radich et al., 2021). These findings suggest that second-line 2G-TKIs treatment may offer a favorable TFR compared to imatinib, even beyond the first-line setting. However, few studies have investigated whether switching to low-dose 2G-TKIs can help restore or consolidate deep molecular responses in patients with prior treatment failure or molecular relapses. In our cohort, three patients who had lost MR4.5 during prior imatinib therapy, while maintaining MR4, switched to low-dose dasatinib with the aim of consolidating or re-inducing deeper molecular responses before potential TFR attempts. All three patients successfully re-attained MR4.5 after switching; however, none had discontinued dasatinib during the study period. These findings suggest that low-dose dasatinib may be effective in restoring deep molecular responses in selected patients, although its role in facilitating TFR requires further confirmation through large-scale prospective studies.

In clinical practice, TKI dose reduction is a key strategy for preventing and managing adverse events, improving quality of life, and enhancing compliance (Rousselot et al., 2021; Jabbour et al., 2022; Li et al., 2025). Management of adverse events was another critical focus of this study. Among the thirteen patients who were intolerant to their TKIs in this study, dose-reductions of their TKIs were initially considered before switching to low-dose dasatinib; however, most adverse events remained unresolved, and the majority of those patients (84.6%) failed to achieve MMR. Therefore, switching to low-dose dasatinib was recommended for those patients to mitigate adverse events and to improve treatment response. Adverse events were alleviated or resolved in most patients after switching to low-dose dasatinib, including those with prior edema, pleural effusion, or hepatic dysfunction. In Alessandra Iurlo’s study, low-dose ponatinib was also shown to be a safe treatment option for patients who were intolerant to other TKIs (Iurlo et al., 2020). These outcomes support the role of TKI dose reduction in improving tolerability without compromising treatment efficacy.

In conclusion, our study suggests that low-dose dasatinib may be a feasible option for selected patients with relatively low BCR::ABL1 transcript levels, particularly those with suboptimal response or intolerance to prior TKIs, as well as those seeking to consolidate deeper molecular responses before a potential TFR attempt. Importantly, close and regular molecular monitoring is essential during low-dose therapy to ensure timely assessment of response and to guide dose escalation when necessary. However, its role in facilitating successful TFR requires further validation through large-scale prospective studies. Despite these promising findings, several limitations should be acknowledged. This was a retrospective, single-center study with a relatively small cohort. Patients enrolled in the study voluntarily received low-dose TKI therapy. Patients concerned about the efficacy of low-dose dasatinib and those with financial challenges did not receive this treatment, potentially introducing selection bias. Among the 53 patients, 13 had prior TKI intolerance, and only three were treated to re-induce a deeper molecular response before a potential TFR attempt. Only descriptive statistical analyses and intergroup comparisons of treatment responses were performed. Multivariate analysis to identify predictors of molecular responses after dose adjustment could not be conducted. Additionally, incomplete BCR::ABL1 kinase domain mutation data are a limitation of this study. Although most patients underwent mutational testing and no dasatinib-resistant mutations were identified in the available reports, incomplete original data warrant cautious interpretation of the findings in patients with prior TKI failure. Future prospective studies with larger cohorts are warranted to validate these findings.

## Data Availability

The original contributions presented in the study are included in the article/Supplementary Material, further inquiries can be directed to the corresponding author.
