# Supplementary material for: Low-dose dasatinib in second-line therapy or beyond for the treatment of chronic-phase chronic myeloid leukemia: a real-world cohort study
Source: Front Pharmacol. 2026 Jun 18;17:1734319. doi: 10.3389/fphar.2026.1734319 (PMC13323030; doi:10.3389/fphar.2026.1734319)
Supplement: Supplementary file 1 [file Table1.docx]

Supplementary Table 1. Parametric and non-parametric presentation for continuous variables regarding patient characteristics, TKI treatment, and molecular responses before switching to low-dose dasatinib

| **Continuous variables** |  |
| --- | --- |
| Age at diagnosis, years |  |
| Median (IQR) | 39 (27-50) |
| Mean±SD (Min-Max) | 38.9±16 (7-70) |
| Age at switching to low-dose dasatinib, years |  |
| Median (IQR) | 42 (33-51.5) |
| Mean±SD (Min-Max) | 41.5±14.6 (14–72) |
| Duration of MMR before switching to low-dose dasatinib, months |  |
| Median (IQR) | 23.5 (7-34) |
| Mean±SD (Min-Max) | 29.8± 33.8 (1-144) |
| Duration of MR4 before switching to low-dose dasatinib, months |  |
| Median (IQR) | 12 (3.5-72.5) |
| Mean±SD (Min-Max) | 32.8±53 (3-127) |
| Time to achieve MMR before switching to low-dose dasatinib, months |  |
| Median (IQR) | 14.5 (6.25-21.5) |
| Mean±SD (Min-Max) | 16.25±11.2 (4-45) |
| Time to achieve MR4 before switching to low-dose dasatinib, months |  |
| Median (IQR) | 37 (10.5-59.5) |
| Mean±SD (Min-Max) | 25.4±26.1 (9-72) |
| Duration of TKI treatment before switching to low-dose dasatinib, months |  |
| Median (IQR) | 30 (4.5-60) |
| Mean±SD (Min-Max) | 42.9± 50 (0.3-190) |
| Follow-up of low-dose dasatinib, months |  |
| Median (IQR) | 21 (13.5-27) |
| Mean±SD (Min-Max) | 21.5±11.4 (3-65) |

IQR: interquartile range; SD: standard deviation.
